# Facultative Hyperparasitism: Extreme Survival Behaviour of the Primary Solitary Ectoparasitoid, *Dinarmus basalis*

**DOI:** 10.1673/031.010.10101

**Published:** 2010-07-09

**Authors:** Danielle Rojas-Rousse

**Affiliations:** Institut de Recherches sur la Biologie de l'Insecte, UMR CNRS 6035, Faculté des Sciences, 37200 Tours, France

**Keywords:** *Callosobruchus maculatus*, *Eupelmus vuilleti*, *Eupelmus orientalis*, *Vigna unguiculata*, Bruchidae, intraspecific, interspecific discrimination, intraspecific competition, cleptoparasitic behaviour

## Abstract

This study investigated the egg-laying behaviour of ectoparsitoid, *Dinarmus basalis* Rondani (Hymenoptera: Pteromalidae), females when faced with a prolonged deprivation of suitable hosts leading to extreme ‘oviposition pressure’. The egg-laying behaviour of virgin *D. basalis* females was tested with *Callosobruchus maculatus* (F.) (Coleoptera: Bruchidae) hosts previously parasitized by the conspecific females in which the developing larvae had reached the last larval instar (L5) or pupae. The hyperparasitism did not prevent the occurrence of superparasitism, but only one *D. basalis* egg from a hyperparasitized *D. basalis* L5 larvae reached the adult stage due to the solitary behaviour of the *D. basalis* larvae. Under these experimental conditions, 60.78% of the *D. basalis* adults emerging from larvae were miniaturized due to the depletion of host resources.

## Introduction

In West Africa (Niger, Burkina Faso, Benin), the solitary ectoparasitoid, *Dinarmus basalis* Rondani (Hymenoptera: Pteromalidae), and its sympatric species *Eupelmus vuilleti* Crawford and *E. orientalis* Crawford (Hymenoptera: Eupelmidae) parasitize the larvae and pupae of *Callosobruchus maculatus* (F.) and *Bruchidius atrolineatus* (Pic) (Coleoptera: Bruchidae) which develop inside the seeds of the cowpea, *Vigna unguiculata* (L) Walp, (Fabales: Fabaceae). After harvesting, these seeds are stocked in granaries where successive generations of bruchids develop, fluctuating in space and time. A survey of the hymenoptera population shows that the most abundant species at the beginning of storage is *E. orientalis* (72%), while *E. vuilleti* and *D. basalis* account for 12% and 16% respectively (Ndoutoume-Ndong and Rojas-Rousse 2007). The *E. orientalis* population decreases gradually during storage, disappearing completely within two months, the majority of which escape directly from the storage structures (Ndoutoume-Ndong and Rojas-Rousse 2008). However, *D. basalis* and *E. vuilleti* have been found coexisting for several months in these structures which form uniform and relatively closed habitats, resulting in inter and/or intraspecific competition (Lammers and van Huis 1989; Monge and Huignard 1991).

The coexistence of *D. basalis* and *E. vuilleti* is based on a counter-balanced competition, i.e. on two opposing behaviours (Zwölfer 1979; van Alebeek et al. 1993). This strategy implies that the females of the competitive species have interspecific discrimination capacities. In fact, *D. basalis* females lay fewer eggs in the presence of *E. vuilleti* females or in hosts parasitized by them (van Alebeek et al. 1993). In contrast, *E. vuilleti* females have developed an aggressive strategy, concentrating their ovipositions on hosts already parasitized by *D. basalis,* killing the *D. basalis* eggs and/or neonatal larvae by stinging them (van Alebeek et al., 1993). Host discrimination involves the detection of external and/or internal cues at the host site. External cues can be detected more rapidly than internal ones and over a longer period since they can be picked up continuously while foraging (Hoffmeister and Roitberg, 1998).

This competitive strategy of taking advantage of resources foraged by heterospecific individuals is a characteristic feature of cleptoparasitism (Sivinski et al. 1999; Jaloux and Monge 2006). The increased encounter rate with parasitized hosts and cleptoparasitic efficiency appears to be based on the detection of two independent signals (Jaloux et al. 2004; Jaloux and Monge 2005, 2006). First, olfactory detection of Dufour gland hydrocarbons, left by the *D. basalis* female on the cuticle on the surface of the seed, allows a seed which has been visited or exploited by *D. basalis* to be recognized. Secondly, detection of the proteinaceous substance produced by the *D. basalis* venom gland and deposited on the edge of the hole drilled through the cotyledon to reach the host indicates that the host has probably been exploited, triggering the cleptoparasitic behaviour. Because this protein is not volatile, it is probably detected through antennal or oral contact chemoreceptors (Jaloux and Monge 2006).

Internal stimuli have poor accessibility but are the most reliable indicators of previous parasitism. The study of intraspecific competition between *D. basalis* females shows that host discrimination is achieved through the perception of cues inside the seed, because it is only when the females have probed the host chamber with their ovipositor that they decide to accept or reject a host for oviposition (Gauthier 1996, Gauthier et al. 2002). In this species, host discrimination is expressed through two mechanisms acting independently (Gauthier et al. 1996). First, a time-dependent process: the deterring oviposition factor(s) can be perceived by the wasp after the first oviposition, reaching a maximum activity with a 24-h-old embryo; secondly, a hostquality indicator process comes into play after a host has been parasitized for 48h (Gauthier et al. 1996). During this time, a transfer of chemical information from the egg to the surface of the host occurs (Gauthier et al. 1996; Gauthier et *a*l. 2002). The gradual deterrent effect supports the hypothesis of substances released by the egg in the course of its embryonic development. One question arising from these observations concerns the means by which the oviposition deterring effect is transferred from the egg to the host (Gauthier and Monge 1999 a).

Faced with hosts offering its offspring little chance of survival, *D. basalis* females lay a few eggs and resorb the others; egg resorption is a transitory process which ceases after 5 days if there is a return to favourable conditions (Gauthier 1996; Gauthier and Monge 1999 b).

However, in the Sudano-Sahelian zone of Burkina Faso and in the Guinean zone of Togo, the biological control of bruchids by releasing *D. basalis* females in leguminosae *V. unguiculata* granaries leads to unfavourable conditions at the end of the storage period due to substantially reduced numbers of the bruchid *C. maculatus* following the development of successive generations of *D. basalis* (Sanon et al. 1998; Amevoin et al. 2007). In this extreme situation, *E. vuilleti* and *E. orientalis* females, living sympatrically with *D. basalis,* are able to express facultative hyperparasitism when confronted by hosts parasitized by conspecific or heterospecific females (Rojas-Rousse et al. 1999; Rojas-Rousse et al. 2005). Facultative hyperparasitism involves the development of the progeny as either primary or secondary parasitoids (Sullivan 1987). In fact, facultative hyperparasitism is a very aggressive behaviour of females that sting and kill the developing primary parasitoid (L5 larvae or pre-pupae or pupae) before ovipositing on it. For *E. vuilleti* females the facultative hyperparasitism can be considered as an extreme expression of cleptoparasitism (expressed only towards eggs and/or neonatal larvae of primary parasitoids) (van Alebeek et al. 1993; Leveque et al. 1993; Jaloux et al. 2004, 2005; Rojas-Rousse et al. 2005).

At the end of the storage period in granaries, because the development of successive generations of *D. basalis* leads to a reduced number of unparasitized hosts and an increased number of parasitized hosts, *D. basalis* females might have to forage among hosts that offer their offspring little chance of survival. To understand the consequences of these particular environmental conditions on the population of *D. basalis,* this study investigated the egg-laying behaviour of *D. basalis* females when faced with a prolonged deprivation of suitable hosts leading to extreme ‘Oviposition pressure’. The egg-laying behaviour of virgin *D. basalis* females was tested with hosts parasitized by conspecific females in which the developing primary parasitoid larvae had reached the last larval instar (L5) or pupae stage. By this time the phytophagous host has been almost entirely consumed by the primary developing parasitoid larva (Rojas-Rousse et al. 2005). Under these experimental conditions of low quality host patches, we investigated the egg-laying behaviour of *D. basalis* females and their ability to develop at the expense of their conspecific larvae, i.e to hyper-parasitize.

## Materials and Methods

### Insect stocks

Bruchid and parasitoid stocks were derived from *C. maculatus* and *D. basalis* adults emerging from cowpea cultures (*V. unguiculata*) at the end of the rainy season in the Niamey region. *C. maculatus* is a common pest that develops inside cowpea seeds, concealed from the parasitoid females.

In the laboratory, bruchids and the primary parasitoids were mass-reared in climatecontrolled rooms under conditions close to those of their area of origin: 12:12 L:D, 23– 33° C and 40% RH.

The strain of *C. maculatus* was maintained by placing males and females (50 pairs) in rearing boxes containing 300 cowpea seeds. The females laid eggs on the seeds, and the neonate larvae perforated the coat. The four larval stages and the pupal stage were completed within the seed.

For primary parasitoid rearing, hundreds of 1 or 2 day-old adults of *D. basalis* were placed in transparent cages (25*30*40 cm) in presence of 200 cowpea seeds containing L4 larvae or pupae of *C. maculatus.* Parasitoids were provided daily with a sucrose saturated cotton roll fixed in the middle of the cage. After 2 days the seeds, parasitized or not, were removed from the cages. Primary parasitoid adults emerged from the parasitized seeds after 12–15 days for *D. basalis* The parasitoid females used in the experiments were isolated in Petri dishes and fed with a sucrose solution.

### Experimental methods

All the experiments were carried out in the laboratory. Translucent gelatine capsules were used that mimic the bruchid pupal chamber, the size and shape being replicated by using both parts of the capsule (Cortesero and Monge 1994; Damiens et al. 2001; Jaloux et al. 2004). This system allows development to occur normally.

### Activation of oogenesis of *D. basalis* virgin females during their first four days of life, production of paralysed hosts, *D. basalis* L5 larvae and pupae males

Immediately after emergence, the *D. basalis* virgin females were put into groups of eight in small cylindrical Plexiglass boxes (250 cm^3^) and provided daily with 16 cowpea seeds each containing one *C. maculatus* L4 larva or pupa primary host until the evening of the fourth day. Egg production reaches a peak on the fourth day of egg-laying and remains constant until the females are 8 days old (Gauthier 1996; Gauthier and Monge 1999 a).

The seeds were removed every day and stored until the terminal developmental phase of *D. basalis* (last L5 larval stage and /or young pupae) on the 7^th^ ± 1 day after egg-laying (Damiens et al. 2001). The seeds were opened to isolate stung and paralysed *C. maculatus* hosts, the L5 larvae and young pupae of which were presented to the *D. basalis* virgin females during the choice tests.

### Hyperparasitism choice between parasitized *D. basalis* L5 larvae and young pupae

To produce extreme oviposition pressure, the *D. basalis* virgin females received no hosts from the fourth to eighth day of life. These virgin females, ‘conditioned’ by deprivation of suitable hosts for 4 days, were used in the hyperparasitism tests.

The *D. basalis* L5 larva or pupa was confined in a transparent cell, the same size and shape as the lodge of a bruchid larva in a seed, and with holes drilled on the surface to simulate the bruchid larval gallery providing access to the host.

Eight ‘conditioned’ virgin *D. basalis* females were kept in a small cylindrical Plexiglass box (250 cm^3^) with eight *D. basalis* hosts put singly into gelatine capsules containing alternately one *D. basalis* L5 larva or one young pupa (total per box: four L5 larvae and four young pupae). Egg-laying was observed every day for 4 days (total in 4 days: 16 L5 larvae and 16 young pupae). Six sets were completed (total: 16x 6 L5 larvae and 16x 6 young pupae). These experiments were carried out under the same climatic conditions as those used for rearing bruchids and parasitoids.

### Choice between non-stung (healthy) and stung-paralysed *C. maculatus* hosts

*D. basalis* can distinguish pupae from L5 larvae by their physiology and by the texture of their integument: soft in larvae and chitinised in pupae. Before egg-laying, *D. basalis* females inflict a sting which has a paralysing action on the host. However, as the females' decision to accept or reject a host is based on the perception of cues inside the seed when they probe the host chamber with their ovipositor, we hypothesized that *D. basalis* females could be deluded about the quality of the host stage (Gauthier et *a*l. 2002). To test this hypothesis that hosts can be rejected due to their immobility after the paralysing sting, the egg-laying behaviour of the ‘conditioned’ virgin females exposed to primary healthy and stung-paralysed *C.* *maculatus* hosts is examined. The same experimental method described above were used, since the *C. maculatus* stungparalysed L4 larvae hosts would be easy to identify due to their immobility and melanized scars (Rojas-Rousse et al. 1995).

These experiments were carried out under the same conditions as those used for choice between *D. basalis* L5 larvae and young pupae. Egg-laying of virgin *D. basalis* females (4 days old) was observed for 2 consecutive days (total in 2 days: 8 stung *C. maculatus* and 8 non-stung *C. maculatus* L4). Seven sets were studied (total: 8 × 7 stung L4 larvae, and 8 × 7 healthy *C. maculatus* L4 larvae). The experiments were carried out under the same climatic conditions as those used for rearing bruchids and parasitoids.

#### Development of eggs laid on hyperparasitized hosts

The number of eggs laid by virgin *D. basalis* females on *D. basalis* hosts was noted, and the hyperparasitized host + eggs were placed in a cell in a Plexiglass sheet closed by a Plexiglass cover-slide until emergence of the hyperparasitoid adult male. This developmental chamber has already been used successfully for developing parasitoids (Darrouzet et al. 2003). As only virgin *D. basalis* females were used, when more than one egg was laid per host, the larval competition that occurred did not affect the final sex-ratio because reproduction was by arrhenokotous parthenogenesis and consequently only male eggs were involved.

#### Statistical analysis

The chi-square test was used to test the homogeneity of the egg-laying behaviour of *D. basalis* females between the data sets. If homogeneity was accepted, all the data sets could be pooled.

To evaluate the hyperparasitism behaviour of *D. basalis* females, the observed numbers of hyperparasitized and non-hyperparasitized L5 larvae were compared with those theoretically expected under the null hypothesis, whereby no preference would be shown by the egg-laying females. According to this null hypothesis, the theoretical probability of hyperparasitism was 1/2. The same method was used to demonstrate the behaviour of the females exposed to non-stung (i.e. healthy) and stung-paralysed *C. maculatus* hosts. The eggs laid on each type of hyperparasitized host were counted and compared using Student's t-test.

### Results

#### Choice between *D. basalis* L5 and pupae hosts

In each data set studied, some *D. basalis* L5 hosts presented at the beginning of the test reached the pupal stage, and the egg-laying distribution indicated that the *D. basalis* pupae were not hyperparasitized (Table 1).

**Table 1.**
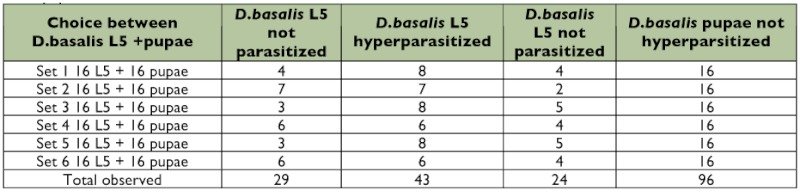
Comparison of hyperparasitism behaviour of virgin *D. basalis* females exposed to *D. basalis* L5 larvae hosts and *D.* *basalis* pupae hosts

**Table 2.**
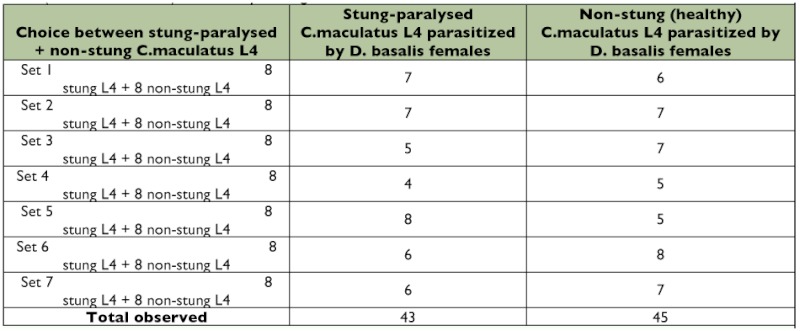
Comparison of egg-laying behaviour of virgin D. basalis females exposed to C. maculatus primary paralysed hosts (i.e., immobile hosts), and healthy moving hosts.

Some of the *D. basalis* L5 hosts were hyperparasitized (Table 1). The null hypothesis was tested that there would be no difference in the proportion of hyperparasitized and non-hyperparasitized *D. basalis* L5 hosts between the six data sets. Since this hypothesis of homogeneity was confirmed, the six data sets were pooled (χ^2^ calculated = 3.65: α = 0.05, χ^2^_ddl 5_ = 11.07).

To evaluate the behaviour of *D. basalis* females exposed to L5 hosts, the numbers of hyperparasitized (N = 43) and nonhyperparasitized (N = 29) hosts observed were compared with those theoretically expected under the null hypothesis. Under these conditions, *D. basalis* females hyperparasitized as many L5 hosts as they avoided (χ^2^ calculated = 2.72: α = 0.05, χ^2^_ddl 1_ = 3.84). These results indicate only that the *D. basalis* females were able to lay on their last stage larvae.

#### Choice between primary non-stung (healthy) and stung-paralysed *C. maculatus* hosts

To test whether *D. basalis* pupae hosts were avoided due to their immobility, *D. basalis* females were presented alternately with stung-paralysed and non-stung (i.e., healthy) primary *C. maculatus* L4 hosts. They were able to lay eggs on both categories of hosts (Table 2).

The null hypothesis was tested whereby no difference of parasitism would be observed between the seven data sets. As this hypothesis of homogeneity was confirmed, the seven data sets were pooled (χ^2^ calculated = 1.53: α = 0.05, χ^2^_ddl 6_ = 12.59).

To evaluate whether *D. basalis* females showed a preference for one or other type of primary host, the numbers of parasitized and avoided hosts were compared with those theoretically expected under the null hypothesis (i.e., 44 parasitized and avoided *C. maculatus* L4 hosts: 43 + 45/2) (Table 2). The observed number of primary hosts (healthy or stung) parasitized by *D. basalis* females was not significantly different from the theoretically expected number (χ^2^ calculated = 0.19: α = 0.05, χ^2^_ddl 1_ = 3.84). Thus, when *D. basalis* females could choose between non-stung (healthy) or stungparalysed *C. maculatus* L4 larvae, they parasitized both categories equally.

**Figure 1.**
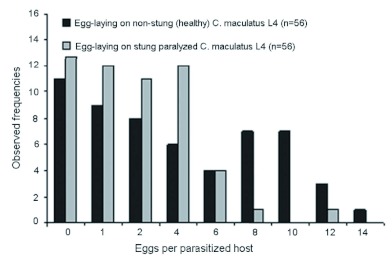
Distribution of eggs laid by *Dinarmus basalis* virgin females exposed to moving, i.e., healthy *Callosobruchus maculatus* primary hosts, and non-moving, i.e., stung-paralyzed *C. maculatus* hosts (previously stung by *D. basalis* females). When there was more than one egg per host, the host was superparasitized. High quality figures are available online.

However, analysis of the eggs laid per host showed that 51.8% (29/56) of stung-paralysed primary hosts, and 64.28% (36/56) of the healthy primary hosts presented, were superparasitized (i.e., more than one egg laid per host) (Figure 1). The percentage of superparasitized hosts did not differ significantly between the two types of primary host presented alternately to *D. basalis* females (*t*-test for frequencies comparison: *t* = 1.02, significance level α = 0.05, *t*_[.05] ∞_ = 1.96). However, on average, *D. basalis* females laid significantly more eggs per healthy primary host than per stung-paralysed primary host: 4.25 ± 1.05 and 2.04 ± 0.29 respectively (mean number of eggs laid ± 95% confidence interval); (Student's test: *t* = 3.65 significance level α = 0.05 *t*_[.05] ∞_ = 1.96).

**Table 3.**
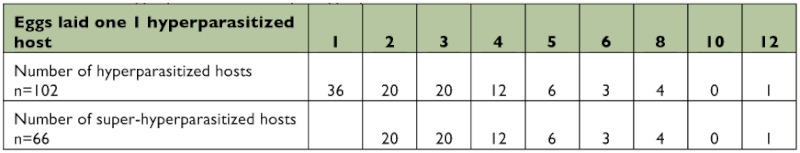
Number of hyperparasitized and super-hyperparasitized hosts

#### Development of eggs laid on hyperparasitized *D. basalis* L5 hosts

The individual development of 102 hyperparisitized *D. basalis* L5 hosts was observed in the Plexiglass cells. Since one to twelve eggs were laid per hyperparasitized *D. basalis* L5 host, hyperparasitism did not prevent the occurrence of superparasitism (Table 3). Although only one egg per host reached the adult stage due to the solitary behaviour of the *D. basalis* larvae, under our experimental conditions we observed that 64.7% of *D. basalis* L5 hosts were superhyperparasitized (66/102) (Table 3). Under these experimental conditions, egg development occurred in three possible ways. First, 60.78% of the hyperparasitized *D. basalis* L5 hosts (62/102) reached the miniaturized hyperparasitoid adult male stage. Second, the eggs hatched but the neonatal hyperparasitoid larvae died allowing the *D. basalis* L5 host to reach the adult stage (i.e., the primary parasitoid adult); this was observed in nine hyperparasitized hosts (9/102). And finally, the eggs laid on 31 hyperparasitized *D. basalis* L5 hosts died during embryonic development (31/102).

### Discussion

Parasitoids use a large number of physical and chemical cues when they forage for hosts, and many of them mark the host patch, the host substrate, and/or the host itself (Godfray 1994; Quicke 1997). These marks may enable females to discriminate between unexploited and previously exploited resources, and also inform other females, conspecifics or heterospecifics, about the presence of a possibly superior competitor (Roiteberg and Mangel 1988). In this way, *D. basalis* parasitoid females are able to discriminate the quality of their host, but detailed behavioural observations show that this host discrimination is based on internal cues, and the lack of evidence of external marks is unexpected (Gauthier et al. 2002). For *D. basalis* females, host quality (healthy or parasitized 24h or 48h beforehand) does not affect the attractiveness of seeds in which hosts are concealed (Gauthier et al. 2002). It is only when the *D. basalis* female has drilled the cotyledons of the seed to reach the host chamber and has probed this chamber with her ovipositor that the decision is made whether to accept or reject the host for oviposition (Gauthier et al. 2002). These internal cues have low accessibility but are the most reliable indicators of previous parasitism and of the age of parasitic instars (Gauthier 1996). However, *D. basalis* females exhibit a wide range of oviposition behavioural plasticity, and host discrimination ability does not always involve avoidance of superparasitism (Gauthier et al. 1996). Under unfavourable conditions, *D. basalis* females are able to resorb the unlaid eggs with no effect on future oviposition because they have a relatively large daily oviposition window compared to the potential number of eggs laid (Gauthier 1996; Gauthier et al. 1996; Gauthier and Monge 1999a).

Superparasitism may sometimes be advantageous when unparasitized hosts are scarce, and/or the neonatal larvae born from eggs of the last oviposition have a better chance of competiting successively against older competitors, and/or the risk of parasitism by conspecific or heterospecific females is high (Visser et al. 1992; Godfray 1994). In this way, when *D. basalis* females superparasitize hosts, the survival probability of the second egg laid has been shown to vary with the age of the first parasite already on the host (Gauthier 1996). In fact, the survival rate of the second parasitoid increases with the interval between ovipositions (Visser et al. 1992; Mackauer 1990).

The use of *D. basalis* in granaries as a biological bruchid control agent leads to an increased number of parasitized hosts creating unfavourable conditions during the storage period (Sanon et al. 1998; Amevoin et al. 2007). These conditions were recreated in the laboratory using *D. basalis* virgin females that did not receive suitable hosts, i.e. primary bruchid L4 or pupae, for four consecutive days. After this extreme oviposition pressure caused by host deprivation, the females were presented with *D. basalis* L5 larvae hosts. Only virgin *D. basalis* were used in these experiments to eliminate all consequences of parthenogenetic reproduction, i.e. number of matings, sex ratio at egg-laying, etc. The *D. basalis* L5 larvae hosts had molted to a passive stage corresponding to the end of the feeding period on the primary host, i.e. bruchid L4 or pupae. Under these experimental conditions, the *D. basalis* virgin females were able to hyperparasitize the *D. basalis* L5 larvae hosts However, future research should investigate the behaviour of inseminated *D. basalis* females under unfavourable conditions, particularly in view of the fact that the progeny of hyperparasitized inseminated *Eupelmus vuilleti* females are largely male (Rojas-Rousse et al. 2005). Since *D. basalis* hyperparasitoid larvae feed externally, no special adaptations may be needed to attack the primary parasitoid (Godfray 1994). Under our experimental conditions, 60.78% of hyperparasitized *D. basalis* L5 larvae hosts became hyperparasitoid miniaturized adult males. Approximately a third of the hyperparasitized *D. basalis* L5 larvae hosts died after being stung by adult females during egg-laying, although the venomous sting generally only induces permanent paralysis and developmental arrest of the host during the development stages of first larvae (Doury et al. 1995). This hyperparasitism corresponds to the facultative hyperparasitism arising from competition between parasitoids for host resources, which is considered as one possible evolutionary pathway leading to obligatory hyperparasitism (Godfray 1994).

The hyperparasitism behaviour did not modify the reproductive behaviour of the egg-laying females because stinging always occurred before egg-laying, although the eggs were laid externally. However, this behaviour did not exclude superparasitism because more than one egg per host could be laid. In fact, 64.7% of the hyperparasitized *D. basalis* L5 larvae hosts were super-hyperparasitized. However, 8.82% of the hosts without stings reached the primary parasitoid male adult stage after all the neonatal hyperparasitoid larvae died during fights.

Under our experimental conditions, the *D. basalis* virgin females could choose between *D. basalis* L5 larvae and pupal hosts. Egg distribution showed that no *D. basalis* pupa host was ever hyperparasitized. Besides their physiological differences, *D. basalis* L5 larvae and pupae can be distinguished by the texture of their integument (soft in the L5 larvae and chitinised in the pupae), which is the basis of mechanical and/or chemical cues perceived by the females' ovipositor at egglaying. These cues could underlie the variability of responses to host quality. Another hypothesis is that it is more difficult for a young hyperparasitoid neonatal larva to become implanted on the chitinised integument of a pupa than on the soft integument of an L5 larva, especially if it has not been immobilised, i.e. paralysed, by the adult female at egg-laying. This behaviour, induced by notable physical differences between the L5 and the pupae, was also observed during the primary parasitism of the *C. maculatus* host. In fact, egg-laying is greater on the larval stages than on the younger non-chitinised and older chitinised pupae (Terrasse and RojasRousse 1986) The immobility of the *D. basalis* pupae alone did not seem to induce the cues favouring their rejection, because when the *D. basalis* virgin females could choose between healthy (i.e. non-paralysed) or paralysed *C. maculatus* L4 larvae, they laid eggs on both categories, although significantly more on the healthy host.

The responses of *D. basalis* females when faced with host deprivation leading to extreme oviposition pressure revealed that they were able to parasitize their own developing primary last instar larvae. Some species of primary parasitoids can be facultative hyperparasitoids, but the hyperparasitism is always interspecific (Strand 1986). The first recorded observation of a facultative hyperparasitoid developing within its own species was *Anaphes victus* (Hymenoptera: Mymaridae), and no other hyperparasitoids are known in this family (Sullivan 1987; van Baaren et al. 1995).

Facultative hyperparasitism is functionally similar to conspecific superparasitism. In solitary parasitoids, conspecific superparasitism can be advantageous if there are high levels of competition, long inter-patch travel times, low quality patches and timelimited resources (van Alphen and Visser 1990). The *D. basalis* females hyperparasitized their own species when they were confronted with a low-quality patch (*D. basalis* L5 larvae or *D. basalis* pupae) and when there was a high level of competition with females who were or had been present in their habitat. With an average success rate of 60%, the facultative hyperparasitism of *D. basalis* females can be seen as highly adaptive when faced with low quality patches. In fact, we observed that the *D. basalis* females were also able to hyperparasitize other species, such as *Eupelmus vuilleti* and *Monoksa dorsiplana* Boucek (Hymenoptera: Pteromalidae) (primary parasitoids of bruchids), but secondary parasitoid adults never emerged (personal observations). However, there are fitness costs associated with this mode of development, because a significant decrease in the size of secondary parasitoids has been observed following the depletion of host resources (Brodeur, 2000).
